# Not All Puppies and Sunshine: How Dog Keepers Cope with Dog-Related Problems in Dutch Society

**DOI:** 10.3390/ani13061038

**Published:** 2023-03-13

**Authors:** Susan Ophorst, Noelle Aarts, Bernice Bovenkerk, Hans Hopster

**Affiliations:** 1 Institute for Science in Society, Radboud University, 6500 GL Nijmegen, The Netherlands; 2 Animal Management, Van Hall Larenstein University of Applied Sciences, Agora 1, 8934 CJ Leeuwarden, The Netherlands; 3 Department of Social Sciences, Wageningen University & Research, 6700 EW Wageningen, The Netherlands

**Keywords:** human–animal relationship, behavior change, coping strategies

## Abstract

**Simple Summary:**

The relationship between humans and dogs is not all puppies and sunshine. Biting incidents, hereditary problems, and other issues can threaten the welfare of humans and dogs. The Dutch government and animal welfare organizations struggle with ways to influence the behavior of (potential) dog keepers. As campaigns tend to focus on risks, it is possible that people will react adversely, because such campaigns evoke negative feelings. People react in different ways to negative feelings, and therefore the focus of this research was to determine the strategies used by dog keepers to cope with negative opinions on (their) dogs. Most coping strategies were found in all groups, but with different manifestations. These differences should be kept in mind when a behavior change in dog keepers is desirable. Attention should be given to communicating with dog keepers in groups, as this research shows that these groups’ boundaries might be far more specific than simply dog keepers. Broad attention on problems with and for dogs can address perceived conflict between beliefs and actions and prompt behavior change. In dialogue with dog keepers, in influential campaigns and in policy formulation, the chances of success are greater if initiators are aware of the strategies that they may encounter.

**Abstract:**

Zoonoses, biting incidents, hereditary problems, and other issues can threaten the welfare of both humans and dogs. The Dutch government and animal welfare organizations seem to have little effect in their campaigns to influence the behavior of (potential) dog keepers, who can experience dissonance when faced with these campaigns and use coping strategies to relieve the dissonance instead of changing their behavior. In this study, in focus group discussions, dog keepers with pedigree dogs, high-risk dogs, foreign shelter dogs, and dogs purchased at puppy farms shared their experiences with opinions on dogs and were confronted with negative opinions on their dogs. The data were analyzed using a coping strategies framework. Most coping strategies were found in all groups, but were used in response to different dilemmas, with different manifestations. These differences should be kept in mind when behavior change in dog keepers is opportune. Special attention should be given to differentiating target groups, as use of the detachment coping strategy suggests that boundaries might be set differently than expected. Broad attention on problems with and for dogs can address perceived dissonance and prompt behavior change. In dialogue with dog keepers, in influential campaigns and in policy formulation, the chances of success are greater if initiators are aware of the strategies that they may encounter.

## 1. Introduction

With over 1.7 million dogs [1] in an area measuring 41,543 km^2^ with a population of 17 million people [2], The Netherlands has a high dog density. This means that every square kilometer in The Netherlands houses on average 41 dogs and 410 humans. With dogs in such close proximity to people, problems may occur relatively more often or be more visible when they occur. A variety of problems can arise, from problems for humans, such as dog bites [3], which can cause severe physical and emotional damage to the victim [4,5], to welfare problems for dogs in general [6] and specifically in pedigree dogs [7,8], to problems for society at large, such as illegal dog trafficking [9] and zoonotic diseases transmitted by dogs [10].

These problems can be related to the dog keeper’s choice of a specific type of dog. The problem of severe dog bites, for example, may be related to high-risk dogs and the dog keeper’s lack of relevant knowledge [11]. Behavioral problems may be related to commercial breeders [12] or a misfit between the dog and its owner, as different types of dogs have different needs [13]. Inherited defects can range from choosing pedigree dogs [14] and illegal dog trafficking, behavioral problems, and zoonoses, to the acquisition of foreign stray dogs [15,16,17]. In all these examples, the value-based *good choice* of the potential owner is crucial. According to Berkman [18], value-based choices are the result of a decision-making process that involves the least effort, either in time, complexity, or distance from the decision maker’s existing point of view.

Making a good choice of dog is highly complex in the absence of facts on what constitutes ‘a good choice’ of dog [19]. One can choose to purchase a puppy or an adult dog from a breeder or an animal shelter. The breeder can be a professional dog breeder or an enthusiastic lay breeder who is incidentally breeding dogs. The shelter can be a Dutch one or one located in, for example, Romania, Russia, Greece, or Spain. One can purchase a pedigree dog, a look-a-like, or a mongrel dog. All these choices have their own particular concerns, thereby adding to the complexity and may contribute to problems with dogs associated with these choices.

NGOs, governmental institutions, and companies run campaigns to stimulate people to make an informed decision on the purchase of a dog, as The Netherlands has no legal limits on the purchase of dogs, such as a license or waiting period. The information sources can confront people with information that challenges their (intended) decision. This will cause cognitive dissonance, a feeling of psychological discomfort when faced with information that deviates from what one has believed to be true [20]. Cognitive dissonance is described by Cancino-Montecinos et al. [21] as fascinating because “it acts as a motivating force in people’s lives”, meant to alleviate emotional reactions. As people strive for emotional equilibrium, they need strategies to create consonance in a dissonant situation [20]. They might perceive the new information as false, consider it not applicable to their own situation, or ignore it all together. In general, people seek desirable outcomes by giving more attention to information that reinforces their pre-existing preferences and by avoiding contradictory information in order to avert cognitive dissonance [22]. Confirmation bias influences the memory, which leads to a higher impact of information that is supportive to the viewpoint of people on their behavior [23]. The normal way for people to deal with the stress that cognitive dissonance causes is to use a coping strategy that helps to justify their choices and behaviors [24]. These strategies may drive people to avoid advice and therefore limit the effectiveness of information campaigns [25,26]. Insight into dog keepers’ coping strategies is therefore valuable in the process of constructing effective interventions to influence dog keepers’ behavior.

Campaigns on dog purchasing generally focus on a large and broad target group: anyone interested in acquiring a dog. There are, however, big differences in how individuals can be addressed effectively [27]. With the variety of people, dogs, and problems, it is hardly conceivable that one intervention or message would fit every situation, and this study will show there is indeed room for improvement in that department. Therefore, in order to identify the mechanisms that make people less sensitive toward interventions such as campaigns by animal welfare organizations or information from the Dutch government, we aim to reveal different coping strategies used by people with different types of dogs to justify their choices.

The research question that needs to be answered in order to achieve insight into dog keepers’ coping strategies is as follows: *What coping strategies for dog-related problems do dog keepers use to justify their choices?*

## 2. Materials and Methods

To answer this research question, a qualitative method was used to provide in-depth information on patterns in dog keepers’ reactions. The research adopted a case-driven interpretive design methodology, with a diversity of dog keepers, relating to a variety of problems surrounding dogs, constituting different cases. Through focus group discussions, we ascertained participants’ reactions to the dilemmas connected with the corresponding dogs and keepers raised by both the facilitator and the participants within the groups. Each case was the subject of two focus group discussions, to allow for iterative contributions and to increase saturation.

The goal of the design was to gain insight into the way in which dog keepers used coping strategies in interactions with dog-related problems. People can experience dissonance in a variety of situations, and the context in which dissonance occurs is likely to be an important factor in terms of how it will be reduced. Cancino-Montecinos et al. [21] encouraged more research on how people reduce dissonance outside laboratory contexts, and consequently this study attempted to do so by adding interaction with others and using the participants’ experiences. This insight was then used to form an overview of coping strategies that may either interfere with (hindering strategies) or contribute to (enabling strategies) the success of current interventions. Suggestions for alternative interventions were derived from this overview.

An example of this process is the imaginary reaction to advice on restricting high-risk dogs: “*He has no clue about dogs!*”. This reaction can be categorized as a misrepresentation, as the source is an animal welfare scientist with publications on dog welfare to his name. This coping strategy probably leads to ignoring information from this source. An intervention can be improved by either profiling the source as ‘one of them’ or attracting a role model from within the target group to deliver the message (see Table 1).

### 2.1. Focus Group Discussions

The choice of focus group discussions to gather data was prompted by the desire to discover as many coping strategies as possible. This interaction could be limited to the facilitator, but adding peers can help to stimulate the reminiscence of experiences as well as unravel differences in coping. Focus group discussions are particularly suitable for exploring subjects that have not yet been thoroughly investigated [28]. In this study, the dog keepers’ coping strategies were looked at interpretatively to generate rich accounts of how people ascribe meaning to and interpret their experience with—opinions on—dog-related problems, with a view to assessing how this might be used to affect policy and change [29].

To ensure that personal attachment to the subject had no influence on the course of the group interviews, a neutral facilitator led the focus group discussions. This facilitator, Dr. Nijkerk, had no connection with this study other than the role of facilitator and had experience with interviewing, particularly with using the laddering interview method [30,31]. An important function of the facilitator was to create a comfortable environment for the participants, to allow them to speak freely [28].

The groups were formed around established dog-related problems: biting incidents, risk of (zoonotic) diseases, health issues in pedigree dogs, and puppy farming. For each issue, two groups were formed with which a focus group discussion was held, thus totaling eight group discussions. To be eligible to be a part of a group, a participant had to have a dog associated with any of these issues, but not necessarily causing problems or suffering from them. To find sufficient participants, an appeal was posted on Facebook and in several dog-related groups on Facebook. The appeal was posted in Dutch to attract Dutch participants, and all participants were confirmed to live in The Netherlands during the focus group interview. Facebook’s snowballing function proved useful in providing enough participants and enough diversity in the groups.

The focus group discussions were held online because of the COVID-19 rules in The Netherlands, which prohibited live gatherings at the time. Therefore, Microsoft Teams was used to record the responses of all participants. All participants had to use both the microphone and camera on their computer. The participants faced the camera during the discussion.

The recordings were stored after transcription, for scientific integrity purposes only, to ensure the participants’ privacy. The participants’ informed consent was particularly important because of the online form in which the group interviews were held and the storage of the recordings.

The focus group discussions were held and transcribed in Dutch. After analysis, the relevant fragments for this paper were translated into English.

The length of each focus group interview varied between 53 min and 92 min.

The number of participants in each group ideally ranged from four to seven, as this number is considered to be manageable in an online setting, provides enough quality in image and sound to allow for analysis, and has enough potential for discussion and reaction. The total number of participants in the focus group discussions was 36.

The groups were formed homogeneously with respect to the problem, as this ensures more freedom of discussion and fewer management problems [32]. Heterogeneity in all groups was sought on sex, age, and residential location. Specific variation was added to the different group categories to include relevant contexts.

Regarding biting incidents, the groups consisted of keepers of high-risk dogs. There is still a lack of research showing that this type of dog actually bites more often than other dogs, but such dogs are associated with the risk of biting and severe injury to people and other animals. It is therefore to be expected that people who keep this type of dog will have developed coping strategies in reaction to public opinion of their dogs. Variation in the number of dogs (of the same type) owned and the age of the dogs was included, as experience can be a factor that influences the coping strategy.

Regarding (zoonotic) imported diseases, the groups consisted of keepers of dogs retrieved from foreign shelters in either Southern or Eastern Europe. In these countries, diseases occur that normally are not present in The Netherlands, such as rabies and echinococcosis. It is unknown how many dogs retrieved from foreign shelters suffer from or are carriers of zoonotic or imported diseases. The possibility of the occurrence of such diseases is, however, greater in foreign shelter dogs than in dogs originating from The Netherlands, thereby making people with this type of dog more likely to be confronted with opinions on this subject, leading to coping strategies. Variation in the organizations through which the participants obtained their dogs was important, because of the diversity in different organizations’ procedures regarding disease prevention.

Regarding health problems in pedigree dogs, the groups consisted of keepers of pedigree dogs. As different breeds encounter different health problems, variation in breeds was needed in the groups.

As illegal puppy traders were not expected to be willing to participate, and their clients are often not aware of illegality, the groups concerning this topic comprised people who reported buying their dog from a puppy trader or a puppy farm. Variation in the number of dogs (acquired in this way) and the dogs’ age was considered desirable.

Participation differed between the cases (see Table 2). Both pedigree dog and high-risk dog keeper groups consisted of five participants. The groups with keepers of dogs from foreign shelters had six and five participants, respectively. Keepers of dogs from puppy farm situations were least attracted to participate; the first focus group discussion was conducted with three participants and the second with only two participants. One individual interview was added, totaling six participants for this case.

Participants applied to be a part of the group that they deemed most fitting. An overlap existed between the pedigree dog group and the high-risk dog group, the high-risk dog group and the foreign shelter dog group, and the high-risk dog group and the puppy farm group. Differences in age, place of residence, and group-specific differences were present in all groups.

All focus group discussions followed four phases:

1. Facilitator encouraged the participants to talk about their relationship with (their) dogs;
2. Facilitator steered towards the way in which participants acquired their dogs and their motivations at the time;
3. Facilitator confronted participants with dilemmas related to their (type of) dog and to other problems related to dogs;
4. Facilitator asked participants how and what their next choice of dog would be and on what considerations they would base their decision.

### 2.2. Analysis

As the aim was to seek out cognitive dissonance, research on the cognitive approach to framing provided a valuable basis for this study. The form in which this study was conducted also allowed for an interactional approach to framing, where cues are exchanged to understand interaction [33]. The set-up of the group interviews allowed for signs of the interactional paradigm through interaction with the facilitator, with group members, and with comments from ‘society’. Attention shifted to the interactional approach, especially where differences existed between the interactions.

In order to recognize coping strategies in this study, dilemmas were distilled from the transcriptions, based on discussions within the groups. When interpretation based on the transcriptions was insufficiently possible, other signs of emotions in the participants including para-verbal and non-verbal, were retrieved from the recordings to allow for interpretation.

To reduce dissonance, people use a variety of dissonance reduction modes [34]. To categorize coping strategies in the group interviews, a combination of strategies defined by Festinger [35], Serpell [36], and Nijland [31] was used as a framework (see Table 3). The basic communication strategies [35] focus on the consonances people attribute to their behavior, and the trivializing and eliminating of the dissonance they experience. The distancing devices [36] indicate the ways people disconnect from their behavior. With the accepting strategies [31] people acknowledge the behavior as problematic. Each contribution of the participants, related to the topic, was screened for the presence of these strategies.

Each focus group discussion was analyzed by two researchers separately. Differences in allocation to a strategy were discussed to achieve interrater reliability [37].

## 3. Results

**Figure 1:**
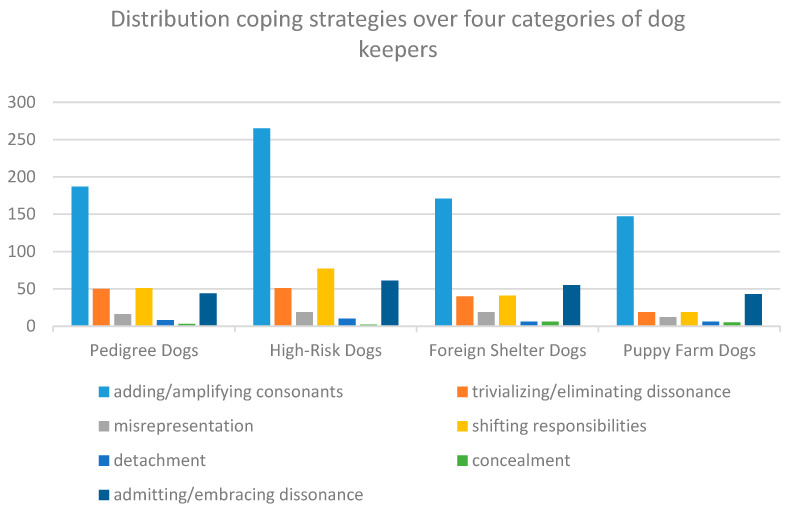
The number of times a coping strategy (Table 3) was found in two focus group discussions of each category of dog keepers.

All strategies were articulated in the focus group discussions, as can be seen in Figure 1. The occurrence of the adding or amplifying consonance strategy was dominant in all groups. The number of occurrences of the other strategies differed somewhat between the groups.

Adding consonance was found frequently in all groups with regard to why the participants chose this type of dog and this particular dog. The detachment strategy was not often used, but occurred in all groups, specifically for participants to distance themselves and/or their own dog from one or more problems specific to their category, creating sub-categories. Other infrequently occurring strategies were misrepresentation and concealment. No patterns were found in these strategies.

The way in which most strategies were used differed between the groups. Different topics were discussed in the groups. In the two groups of the same category however, the topics were largely the same. Patterns of arguments surrounding the different topics are presented below, divided into the different categories of dog keepers.

### 3.1. Pedigree Dogs

Important expressions of consonance used by pedigree dog keepers are the predictability of a pedigree dog (“*you know what you are getting*”), the desire to get the dog as a puppy (“*you make your own dog*”), the unattractiveness of alternatives (“*I know a lot of these imported stray dogs and they are all, no exception, so incredibly scared*”), and the extensive preparation before getting a puppy (“*we searched for a reputable breeder, who does all health testing*” and “*you have to go and see the parents before there are puppies and also visit several breeders before you make a decision*”).

The shifting responsibilities strategy was used by pedigree dog keepers when the health of pedigree dogs was discussed. All participants in the pedigree dog groups viewed the breeder as a highly responsible party in the health of the pedigree dog. Reference was made to the complexity of genetics and the diversity in health problems, both of which make it impossible for a layperson to make proper decisions, thus *shifting responsibility* to the breeder or the breed club. Show judges were also mentioned as responsible for encouraging the breeding of harmful traits in pedigree dogs.

The dissonance involved in the choice of a pedigree puppy was trivialized by emphasizing that trust in the breeder’s ability to avoid hereditary problems trumped the alternative of acquiring a puppy without pedigree: “*As they use pedigree dogs but without testing properly, you have an even bigger risk*”.

The dog’s appearance was mentioned as (an important) part of its attractiveness to pedigree dog keepers. This argument allowed for shame on the part of the participant, as it was mentioned together with a caveat: “*I know it’s stupid, but…*” or “*I’m ashamed to admit it, but*…”, which fits in the admitting dissonance coping strategy.

A form of the detachment strategy was found in these groups when breeds other than their own were discussed. The participants referred to other breeds as problematic and distanced themselves from these breeds, for example: “*Those French Bulldogs with flat faces that keep getting flatter*”.

### 3.2. High-Risk Dogs

The term high-risk dog or tough dogs already raised some discussion on whether or not these dogs posed a higher risk or were that tough, for example: “*He’s a real Millie, not tough at all!*”, with owners detaching themselves from the discussion on these dogs. ‘Power breed’ seemed a good alternative term in the second group: “*Let’s call it power breed*”.

The consonance added as a strategy in these groups featured qualities of the high-risk dogs, such as loyalty to their family and protection. Several participants referred to innate characteristics, originating in the roots of the breed. Characteristics such as aggression toward other dogs were associated with the breeds of dog-fighting origin, and aggression toward people was associated with the breeds originally bred to protect cattle or people. The participants’ effort to raise their dogs properly was also apparent, for example: “*he was distracted easily and it took a lot of training to get that out*”, even though it was not always easy to find a place that welcomed power breeds, for example: “*We were not welcome there, because they said they didn’t want to take that risk with other dogs*”. Specific knowledge about training power breed dogs was advised in order to combine nature and nurture: “*They have an eye for what is possible within the limitations of the breed*”.

Emphasis was placed on the importance of “*knowing what type of dog you are getting*”, especially with these types of dogs. All the management measures taken by the high-risk dog keepers to prevent problems were striking, varying from moving to a remote area or walking the dog in remote areas, to fences, muzzles, and instructing or informing other people. Almost all participants, however, also described incidents where management failed. The reaction to these failures fits the strategy of shifting responsibility.

In the cases where management failed, other people—who did not have the knowledge about how to interact with a high-risk dog—were mentioned: “*but you can only do so much. If I’ve taken every possible precaution and someone keeps throwing the ball in the direction of my dog, it’s out of my hands*”. This shift in responsibility was challenged in one group by a participant who said: “*They don’t choose this type of dog, so they have literally no idea and you can’t expect them to get it*”. To which the response was “*I don’t expect them to get it, as long as they would just listen to me if I warn them. Simple decency*”. Less knowledgeable keepers of high-risk dogs were also held responsible. In both groups, a license to keep high-risk dogs was seen as a viable solution: “*I would be all for a license of some sort*” and “*I don’t think a license is a strange idea at all*”, and this could include specific rules for keeping certain types of dogs, for example: “*Maybe that’s something to put in a license, what the natural freedom of movements is that guard dogs need*”. This license did not have to be just for power breeds: “*I think a license to keep dogs in general wouldn’t be a bad idea*”. Responsibility was also shifted to the people stigmatizing high-risks dogs, particularly the media: “*I think it’s Facebook. When there’s a biting incident with a dog, it’s a Stafford or a Pitbull. When it’s not a Stafford or a Pitbull, it’s a dog. Probably it was a Labrador then, I don’t know*”, and “*I wonder if it isn’t just a media thing. They just look for the most damage or what’s most sensational. If the neighbors’ Dachshund bites, it’s not that interesting*”. Another use of shifting responsibility was seen in these groups, emphasizing the importance of breeding dogs with steady temperaments: “*You have to fix it at the root and that’s the breeders*”.

Breed traits like high sensitivity were recognized as problematic: “*He gets over-stimulated so easily and then he gets that typical Staffy-bark, people with Staffs know what I mean, and then I know that I have to get him out of that situation fast*”*,* and also “*if another dog is picking a fight, they just won’t step down, it’s on*”; these are examples of the admitting/embracing dissonance strategy. Participants for the most part agreed on risks involved with keeping these dogs, divided into risks for people or for other dogs/animals, depending on the breed or type.

The incidents that occurred were in some instances trivialized by emphasizing the relativity of the damage: “*If he really had wanted to fight, he would have done a lot more damage than just a scratch*”. Trivializing was also used when participants talked about the limitations that come with high-risk dog keepers’ management choices. As one of the participants put it, “*It’s a way of life and you get used to it*”.

### 3.3. Dogs from Foreign Shelters

The participants in the foreign shelter dog groups had dogs that they had adopted from shelters in Spain, Greece, Russia, Romania, Hungary, and Bulgaria. Ideas were expressed on differences between dogs from Southern European countries and dogs from Eastern European countries, using the detachment strategy: “*Dogs from Spain often have more experience with living in a house than dogs from Romania or Bulgaria*”, and “*In Eastern Europe, you have a lot of these mountain dogs, Owcharkas, well…you really have to think about if you want that type of dog in a country like The Netherlands. Very different from the Podenco-type dogs from Spain*”. Differences in diseases were also mentioned, with warm-country diseases such as Leishmania and Ehrlichia prevailing in dogs from Mediterranean countries.

One of the striking consonances expressed by the foreign shelter dog keepers was their willingness to adjust their lives to the needs of their dogs, no matter the impact on their own lives. Another important consonance was the conviction that adopting a dog from a foreign shelter was the right choice: “*Adopt, don’t shop*”. Consonance related to the opinion that there were already enough dogs in the world and the situation in foreign shelters was not comparable to that in Dutch shelters: “*They have nice kennels here, in Spain I’ve seen ‘kennels’ made from pallets and binders. It’s no comparison*”, although most of them would happily consider a dog from a Dutch shelter.

In several instances, the participants mentioned not particularly being in favor of adopting a foreign dog or even being opposed to it, admitting to dissonance caused by their decision to adopt a foreign dog. Reasons given for this opposition varied from health risks to behavioral problems in foreign dogs, to the risk that dog trafficking could be disguised this way. These dog keepers claimed that they would have preferred to adopt a dog from a Dutch shelter.

In this context, the shifting responsibility strategy was often used to transfer responsibility for their choice to the Dutch shelters, for example, “*it’s impossible to get a dog when you work. Doesn’t matter how well you have thought it out*”, and “*it’s impossible to get a dog when you’re a student*”. Responsibility was also shifted to mediating organizations. Several times participants mentioned that there was no quality mark for these organizations and that they were not all doing what they should be doing: “*The date of the Leishmania test was disputable, because he was already supposed to be in The Netherlands on that date*”, and “*The character description hardly ever matches the character of the dogs, once they arrive here*”. Knowing mediators personally and having mediators who also mention problems—“*Not all puppies and sunshine*”—evoked trust. The dogs’ behavioral problems were blamed on the situation in the foreign shelters or the way in which they were treated before being housed in the shelter: “*it’s just the mentality over there, how they treat dogs*”.

Trivializing dissonance was found mostly in relation to the decision to adopt a foreign dog, despite reservations. It also occurred in relation to the inconsistencies of the mediating organizations, such as in the story of one participant who discovered that her dog was suffering from Leishmania, but she had not been told this despite the intermediaries having prior knowledge: “*It wasn’t really the fault of the organization. It was more communication between the shelter and the organization*”.

### 3.4. Puppy Farm Puppies

Participants for the puppy farm dog group were hard to come by. Some participants were found through their application to be a part of another group, and some were registered by others, specifically a dog trainer, but were not really sure if they ‘belonged’.

Consonance was expressed by these participants mostly on their preparation before acquiring a dog. They decided that their new dog had to be a puppy, “*because I wanted to influence how he would grow up*”, and “*I have sufficient time to raise a puppy*”. The participants also deliberated on different breeds and character traits. After the preparation, the decision to choose this dog was made quite suddenly, fueled by coincidence, circumstances, and feelings: “*We were orientating for a long time and then, coincidentally, his picture appeared on the opening page of Marktplaats [advertising website]. After being burgled, we didn’t want to wait anymore and it all happened fast*”; several participants claimed that they fell in love with the puppy instantly, and “*That was it, nothing you could do about that*”.

Admitting dissonance was a strategy that all participants used easily: “*Really stupid. I can’t make it any prettier*”, but this conclusion often entailed the ‘past self’. It stretched to the broader subject of preparation in this comment: “*I just didn’t know that at the time and I didn’t look into that at the time. So…*”. It therefore seemed to be a combination of admitting dissonance and detachment from the past self. Dissonance was also admitted by referring to the many warning signs on puppy farming when they were visiting: “*Went home with a different feeling, then we left. It was kind of a sad display*”, “*It was all a bit secretive*”, “*You could tell the mother had a lot of pups, by her nipples*”, “*It didn’t feel right*”, “*We weren’t allowed to see the parents*”, and “*There were about a 100 alarm bells ringing!*”. The participants also expressed remorse about buying puppies at puppy farms: “*We endorse puppy farming by buying them*”.

The responsibility was shifted to the breeders on several occasions: “*They should just tell the truth. Be honest*”, and “*I just didn’t know breeding dogs was a business. I thought all dog breeders were nice people and I didn’t check at all*”. Shifting responsibility could also be found in reactions to what was lacking: “*There should be a quality seal above the door to indicate it’s a good breeder*”.

Trivializing occurred after the participants had admitted to dissonance on the purchase of the dog and then went on to express the contentment that they experienced with their dog: “*But I have her for six months and she’s an absolute doll. Now, I don’t regret it at all*”. One participant trivialized the dissonance with the argument of inevitable uncertainty: “*You never know. Pedigree dogs, shelter dogs, mutts… you just never know*”.

## 4. Discussion

The results of this study clearly show diversity in the subjects addressed by the participants. The use of coping strategies seems to indicate a less diverse pattern. The way in which the participants gave substance to the coping strategies, however, differs between the categories.

The adding consonance strategy was the most found strategy in all categories. However, this strategy was actively found by the facilitator in the second phase of the focus group discussions. Kahneman et al. [38] pointed out that people rationalize their actions after the decision is made. We were therefore interested in these rationalizations by the participants in the different categories, which we found in abundance. As the results were analyzed qualitatively, the impact of this quantitative distortion is not methodologically relevant, but it may have influenced the way in which the data were presented and viewed. It is therefore important to keep this bias in mind.

An interesting division was found between the pedigree dog keepers and the puppy farm dog keepers on one hand, and the high-risk dog keepers and foreign shelter dog keepers on the other. The first two groups added the compatibility of the dogs with their own wishes and needs as consonant, whereas the focus in the other two groups was placed on the compatibility of the keepers to the needs of the dogs. Compatibility of humans and dogs is an important factor for the welfare of both humans and dogs [39]. Emphasis on the needs of one of the parties involved might therefore jeopardize the welfare of the other party. In improving interventions, adding focus on either compatibility with people’s needs or on compatibility with the dog’s needs could be dependent on the target group.

The detachment strategy was one of the least found strategies but was used in an interesting pattern. In all groups, the need to distance oneself from the problematic part of the group at large played an important role in the use of this strategy. In the pedigree dog keepers’ groups, this was found in the distinction between ‘unhealthy breeds’ and the breeds owned by the participants. A distinction can be made in the (perceived) impact of inherited disorders of different breeds [40], although all breeds have challenges in this department. Trivializing the problems of one’s own breed or own dog will probably still occur if the population is divided further, given that a participant with a French Bulldog distinguished between French Bulldogs with a nose and those without. Dog keepers’ unrealistic perception of good health in dogs, established by Packer et al. [41], also attests to this. Segmentation may, however, diminish the use of this strategy and allow for more recognizability of the problem by the target group.

In the high-risk dog keepers’ groups, detachment was found in the distinction between ‘good keepers’ (the participants) and ‘bad keepers’. Knowledge of the breed or type of dog kept and willingness to take management precautions to avoid problems seem to be the factors that distinguish the two groups. Ownership of high-risk dogs is considered a marker for criminal behavior [42]; this could confirm this distinction and add components to the profile of the ‘bad keeper’ as discerned by our participants. As a correlation between these factors and the occurrence, although not exclusively, of biting incidents with these types of dogs seems logical, these factors could be used to target sub-groups of high-risk dog keepers or even be integrated in the license proposed in these groups.

In the puppy farm dog keeper groups, detachment from ‘the younger, ignorant self’ was a distinctive example of this strategy. Participants indicated that they “*didn’t know any better*” and embraced dissonance for past decisions, but from a perspective that they had developed beyond that stage now, thus detaching themselves from their past. This could be a prompt for targeting future dog keepers who have not kept a dog before. As the low participation in this category could indicate, as discussed before, that people have trouble recognizing a puppy farm, more attention could be paid, in future research and campaigns, to knowledge in this area. To complicate the recognizability of a puppy farm, different views exist on what a puppy farm is [43]. It is a commercial dog-breeding facility, but the adequacy or otherwise of the breeding conditions cannot be defined by law only, but also by what people consider to be adequate, and there may very well be a distinction between these views on adequacy. There are also facilities seen as puppy farms where the dogs are not actually bred, but litters are gathered, mostly from a foreign country, and sold from this facility. Buyers and potential buyers are often not aware that the pups have not been bred at the facility [44]. Transparency is key in addressing the problems in this sector.

In the foreign shelter dog keeper groups, this strategy was less prevalent, but could be seen in the distinction between dogs originating from Southern Europe and dogs originating from Eastern Europe. No participant favored dogs from the other source, and both behavioral and physical problems associated with foreign shelter dogs were more ascribed to dogs from the other source. There can be a difference between these groups in terms of health and behavioral risks, or possibly their connection with puppy farming; this would be interesting for future research, as research on this topic [17,45] does not seem to differentiate between different origins.

As we have argued that dog keepers cannot be seen as a homogeneous group regarding the problems raised in relation to dogs, and therefore cannot be addressed in the same way on all topics, the findings support that argument. In this study, we have tried to distinguish between different groups, but the occurrence of the detachment strategy in all groups shows that it is important to look at using even more distinction to overcome the effect of the detachment strategy on arguments. This can be done by addressing dog keepers on a more specific level, e.g., keeper of a brachycephalic breed dog, rather than keeper of a pedigree dog, thereby also allowing for even more specification of the problems related to the dog. It is possible that this coping strategy will nevertheless remain intact, as its—unconscious—purpose could be to avoid behavior change [26]. In information campaigns targeting potential dog owners, it could, however, prove fruitful to try out segmentation of the target group. Another type of intervention that could stem from this result is the use of interpersonal communication instead of, besides, or integrated with, mass media campaigns. The development of computer-mediated communication offers a variety of possibilities to incorporate more dialogue [46].

Another distancing device [36] found to be an important strategy in all groups is shifting responsibility. Several organizations and professional groups were mentioned: dog breeders, breed clubs, and mediating organizations for foreign shelter dogs. Interestingly, all these groups seem to lack clear boundaries to establish the quality of their services or a system to hold them accountable. As suggested in some groups, clear systems for breeding, selling, or mediating dogs, to which the public have easy access, would be an important step in preventing problems. This strategy, however, is also referred to as the denial of responsibility [20], which could be connected to problems with self-reflection [47]. This suggests that attention should be given to encouraging self-reflection by dog keepers, even if other parties share responsibility. Interactional strategies to stimulate dog keepers to engage in dialogue on their presumptions and interests, making these explicit in the process, will prove valuable for this purpose.

Admitting dissonance is another strategy found in all groups. In all groups, this was represented by expressions such as “*one look into his eyes and that was it*”, and “*instantly fell in love*”, and the embarrassment that this evoked in dog keepers. In high-risk dog keepers, admitting dissonance concerned high-risk dogs’ unfavorable behavior. In puppy farm dog keepers, it was about how the purchase of the dog came about. In foreign shelter dog keepers, it was mostly the choice of a foreign shelter dog versus a Dutch shelter dog that caused the dissonance to which they admitted. In pedigree dog owners, the only subject that aroused dissonance, to which they admitted, was the influence of the dog’s appearance. This strategy can be a promising strategy from the perspective of willingness to change. Stone et al. [48] described the reaction of people confronted with past behavior that was contradictory to their current endorsements, whereby they engaged in compensatory behavior to make up for their hypocrisy. Translated to our cases, this would mean that dog keepers could show a willingness to change their behavior in situations in which they are using the admitting dissonance strategy. Admitting and embracing dissonance are coping strategies also found in relation to consumers’ meat-eating choices [31], where these coping strategies were viewed as a way to accept ambiguous situations, thus maintaining the status quo instead of moving toward a change in behavior. If the behavior of admitting and embracing dissonance continues while or after using this strategy, this does not support Stone et al.’s [48] expectation that compensatory behavior would occur. It would be valuable to test this hypothesis in real life, as suggested by Cancino-Montecinos et al. [21], by investigating the intention to perform other behavior when a person is considering the acquisition of a new dog, following up on future decisions about acquiring a dog, or observing the ‘first look’ moment. If admitting and embracing dissonance proves to bode well for future behavior change, its transfer to other desired behavior change, e.g., health or climate-supporting behavior, could be worthwhile. In a previous study [49], an important finding was the absence of Dutch newspaper and Facebook reporting on some of the problems with dogs. In our pedigree dog keeper groups, some participants indicated that they had never encountered negative opinions on pedigree dogs. These participants kept one pedigree dog of a non-health-wise notorious breed as a family dog, with no specific relation to the pedigree, such as breeding or sport. For them, there were no problems with pedigree dogs. If the goal is to get people to think about hereditary diseases, as the arousal of dissonance can be a factor in positive behavior change [48,50], this could be challenging to achieve, given that society at large is not currently arousing dissonance on this subject. Purposely confronting people with an interest in pedigree dogs with risks in relation to past choices might allow for people to come to a more deliberate decision in the future.

### Limitations of the Study

Our study is subject to limitations. All participants in the focus groups still had a dog. In this respect, one could say that they are successful dog keepers, sometimes at considerable cost to themselves, ranging from the management measures for power breeds to adjusting lifestyle for foreign shelter dogs. People who have re-homed their dog or chosen not to have any more dogs in the future may use different coping strategies to cope with their choice. This specific dog-related situation could generate an even broader insight on dog keepers’ coping strategies, as would researching coping with the euthanasia of dogs and several other possible subjects.

Gender bias in this study is possible, because most participants (92%) were female. As gender differences are found on attitudes towards animals [51] differences in coping strategies can be envisioned. Differences in the use of coping strategies between sexes are found [52], although the most striking difference is that women make more use of coping strategies. In a study by Kelly, Tyrka, Price, and Carpenter [53], the adverse effect on women of the use of some copings strategies was found to be larger, while most coping strategies were used by both male and female participants. We set out to attract more male participants, because mixed gender groups tend to improve the quality of discussions [54], and we managed to have a male participant in a pedigree dog group, a high-risk dog group and a foreign shelter dog group. No difference which could be explained by gender bias was noticed between the groups with and without male participants.

In the process of recruiting participants and during the two focus group discussions with high-risk dog keepers, it was clear that the term high-risk dog evoked emotion. The Netherlands does not have any laws prohibiting or regulating certain types of dogs, so there is no legal basis for the term high-risk dog. However, the debate on types and breeds of dogs, associated with the possibility to inflict great physical harm when biting, is current and has had an impulse by the publication of advice by the Dutch Council for Animal Affairs to the Dutch government to compile a list with high-risk breeds [11]. All participants in these focus groups kept dogs that were on the draft of that list, which never came into effect. It is possible that potential participants were repelled by the use of ‘high-risk dogs’ in the recruitment, and those potential participants could have added new coping strategies to the discussion, although the theme of the term ‘high-risk’ still was prominent in both focus groups. In future research, it could prove to be interesting to recruit with different terms, e.g., the term ‘powerbreeds’, which was suggested in one of the focus groups, and establish the difference between participants. To diminish an unwanted effect in this study, in the recruitment of participants the term ‘high-risk’ was always put within quotation marks and often preceded by the words ‘so-called’.

In this study, each category consisted of two groups. It is possible that saturation would have benefitted from more groups per category. Themes from the first groups were incorporated by the facilitator in the second group discussions, but limited new themes occurred in the second groups. In the second group of the puppy farm dog keepers, relatively, the most new themes arose, which can correspond with the low number of participants in these groups. To achieve more saturation in this category, an individual interview was added [55]. As the data provided sufficient ‘thickness’ to gain insight into coping strategies of dog keepers, we consider the saturation satisfactory [56].

The number of participants in the groups could have been higher, as most focus group studies use between six and twelve participants [56]. In this study, it was necessary to do the focus group discussions online instead of face-to-face. Moore, McKee, and McCoughlin [57] found smaller groups to be preferable, as they are more manageable online and the discussions were easier to follow, and the experience in this study confirms that. Conducting the focus group discussions online may have impacted the quality of the discussions, as online discussions tend to have shorter-sentence contributions and address fewer themes [58], although Reid and Reid found a larger variety in ideas in an online environment [59]. This can be a reason for achieving a satisfactory degree of saturation in two groups per category. Another advantage of online focus group discussions is the participation level, which in online discussions is found to be more uniform [60]. This may have avoided unwanted effects found in focus group discussions, such as first-speaker contamination [61].

The groups in the puppy farm dog keepers’ category were too small. Various methods were used to attract more participants: more calls in more Facebook groups and the use of intermediaries. The intermediaries were owners/trainers of dog schools who were asked to contact students who they knew, or had strong reasons to believe, had purchased their dog from a puppy farm. Interestingly enough, these intermediaries reported several times that their students were in denial about the origin of their dogs and reacted negatively or were even offended by the trainer’s request to participate in this category. This could mean that puppy farm dog keepers are not aware of what constitutes a puppy farm, or that they are too ashamed to admit that they purchased their dog there and can be viewed as using the coping strategies of misrepresentation or concealment [36]. These strategies were not often used by the participants, but could be more widespread under non-participants. The negative connotation of puppy farms [62] seems to be corroborated by this reluctance to participate. In future studies, the negative connotation of puppy farms could be taken into account by setting up individual interviews and exploring new ways to contact the proposed dog keepers.

## 5. Conclusions

Coping strategies were used by all participating dog keepers to cope with cognitive dissonance related to their dogs’ problems. The strategies used differed, however, between the keepers of different types of dogs. Adding consonance, trivializing dissonance, shifting responsibility, and admitting dissonance were found in all groups, but were used in response to different dilemmas, with different manifestations. These differences should be kept in mind when behavior change in dog keepers is opportune. Differences in coping strategies call for differences in interventions used to influence the behavior of prospective dog keepers. Special attention should be given to differentiating target groups, as use of the detachment coping strategy suggests that boundaries might be set differently than expected. Broad attention on problems with and for dogs can address perceived dissonance and prompt behavior change. In dialogue with dog keepers, in influential campaigns, and in policy formulation, the chances of success are greater if initiators are aware of the strategies that they may encounter.

## Figures and Tables

**Table 1:** Example of an improved intervention for a target group to preempt interference by a coping strategy.

Target Group	Coping Strategy	Interference	Improved Intervention
High-risk dog keepers	Misrepresentation	Ignoring information	Altering the source
	“He has no clue about dogs”.		Profile the source as ‘one of them’ Attract a role model to deliver the message

**Table 2:** Number of participants per focus group of each case.

Case	Focus Group 1	Focus Group 2
Pedigree Dogs	5	5
High Risk Dogs	5	5
Foreign Shelter Dogs	6	5
Puppy Farm Dogs	3	2

**Table 3:** Framework for categorizing coping strategies based on Festinger [35], Serpell [36], and Nijland [31].

Festinger—*Basic communication strategies*	Adding and amplifying consonance to behavior	“*We searched for a reputable breeder, who does all health testing*”
	Trivializing and eliminating dissonance	“*If he really had wanted to fight, he would have done a lot more damage than just a scratch*”
Serpell—*Distancing devices*	Misrepresentation	“*Then you just can’t say no anymore*”
	Shifting responsibilities	“*Breeders should just tell the truth. Be honest*”
	Detachment	“*I just didn’t know that at the time and I didn’t look into that at the time. So…*”
	Concealment	“*Well… that’s not a discussion for now…*”
Nijland—*Accepting strategies*	Admitting and embracing dissonance	“*I know it’s stupid, but…*”

## Data Availability

Data available on request due to restrictions, e.g., privacy or ethical. The data presented in this study are available on request from the corresponding author. The data are not publicly available in deference to participants’ privacy in accordance with the approval of the Ethics Committee.
